# Impact of GST thickness on GST-loaded silicon waveguides for optimal optical switching

**DOI:** 10.1038/s41598-022-13848-0

**Published:** 2022-06-13

**Authors:** Jorge Parra, Juan Navarro-Arenas, Miroslavna Kovylina, Pablo Sanchis

**Affiliations:** Nanophotonics Technology Center, Universitat Politècnica de València, Camino de Vera s/n, 46022 Valencia, Spain

**Keywords:** Integrated optics, Silicon photonics

## Abstract

Phase-change integrated photonics has emerged as a new platform for developing photonic integrated circuits by integrating phase-change materials like GeSbTe (GST) onto the silicon photonics platform. The thickness of the GST patch that is usually placed on top of the waveguide is crucial for ensuring high optical performance. In this work, we investigate the impact of the GST thickness in terms of optical performance through numerical simulation and experiment. We show that higher-order modes can be excited in a GST-loaded silicon waveguide with relatively thin GST thicknesses (<100 nm), resulting in a dramatic reduction in the extinction ratio. Our results would be useful for designing high-performance GST/Si-based photonic devices such as non-volatile memories that could find utility in many emerging applications.

## Introduction

Similarly to micro- and nano-electronics, silicon has become the mainstay material for developing photonic integrated circuits (PICs). Silicon photonics (SiPh) has been established as an appealing technology for a wide range of applications in the fields of telecom and datacom^1^, quantum computing^2^, or LiDAR^3^, to name a few. However, the active properties of silicon either based on nonlinearities^4^ or electro-optic effects^5^ are rather modest, thus imposing several trade-offs such as compactness and bandwidth. In such a way, there is a compelling interest in the integration of complementary metal-oxide-semiconductor (CMOS) compatible materials featuring a large refractive index modulation at telecommunication wavelengths to overcome those limitations and provide new functionalities to the SiPh platform.

In this context, a plethora of new materials have been proposed in recent years, namely: ferroelectrics, by relying on Pockels effect for enabling high-speed and loss-less refractive index modulation such as barium titanate (BTO)^6^ and lithium niobate (LiNbO$_{3}$)^7^; two dimensional (2D) materials, which exploit their large electro-refractive index such as graphene^8^ and transition metal dichalcogenides (TMDs)^9^; transparent conducting oxides (TCOs) such as indium tin oxide (ITO) by leveraging the strong light-matter interaction (LMI) provided by the epsilon-near-zero (ENZ) regime^10–12^; and phase-change materials (PCMs) such as vanadium dioxide (VO$_{2}$)^13^ and chalcogenides^14^ that stand out for enabling ultra-compact devices thanks to the dramatic change in the refractive index together with, in chalcogenides, a reversible non-volatile response at room temperature.

Ge$_{2}$Sb$_{2}$Te$_{5}$ (GST) is one of the most widely used among the different chalcogenide compounds^15^. At telecom wavelengths, the refractive index of GST typically changes in the order of $\Delta n \sim 2-3$ and $\Delta \kappa \sim 1$ in the real and imaginary parts, respectively, between the amorphous (a-GST) and crystalline (c-GST) state^16–18^. To undergo the crystallization, the GST needs to be heated above $\sim 180^{\circ }$C, while for the amorphization, the material must reach the melting temperature ($\sim 650^{\circ }$C) followed by a rapid cooling ($>1-10^{\circ }$ C/ns) to produce the melt-quench^19^. Otherwise, the GST is likely to be recrystallized. The phase change can be achieved on-chip by self-heating the material with optical pulses and evanescent-field coupling^20^ or placing a microheater near the GST^21,22^. On the other hand, the switching between both states can be achieved with sub-nanosecond times^23^, sub-picojoule energies^24^, and up to $10^{15}$ cycles^25^.

Such appealing properties have led to developing high-performance and new kinds of waveguide-based photonic integrated devices such as modulators^26–30^, pass polarizers^31^, photonic memories^32–37^, reconfigurable devices^38–40^, switches^41–47^, or synapses^48^. Thereby, enabling new applications based on non-von Neumann architectures such as arithmetic^49^ and logic^50^ operations, in-memory computing^51^, neuromorphic computing^52,53^, or photonic tensor cores^54^. Moreover, the non-volatile characteristic of GST would be desirable for achieving silicon photonic devices with ultra-low power consumption^55^.

A waveguide loaded with a thin patch of GST is the basis of most GST-based photonic devices, which usually exploit the large change in the absorption. Therefore, minimizing insertion losses while maximizing the extinction ratio is the target for such absorption-based devices. In this context, the thickness of the GST layer directly impacts the optical performance of GST/Si waveguides. Compared to silicon, GST exhibits a larger real part in its refractive index in both states. Hence, the light can be confined and guided (with loss) in smaller cross-sections than their silicon counterparts. However, as we demonstrate in this work, the coupling and the guiding interplay between silicon and GST can reduce the expected optical performance in contrast to with the assumption of single-mode operation. Therefore, the analysis of the GST thickness in such terms is necessary to obtain optimal optical switching and provide a more in-depth insight into the optical behavior of such hybrid waveguides.

We demonstrate through numerical simulation and experiment that using relatively thick GST layers does not imply an enhancement of the optical performance. In fact, this is reduced compared to thinner layers because higher-order modes with a low extinction ratio are excited when the GST is crystalline.

## Results

### Description of GST-loaded silicon waveguides

**Figure 1:**
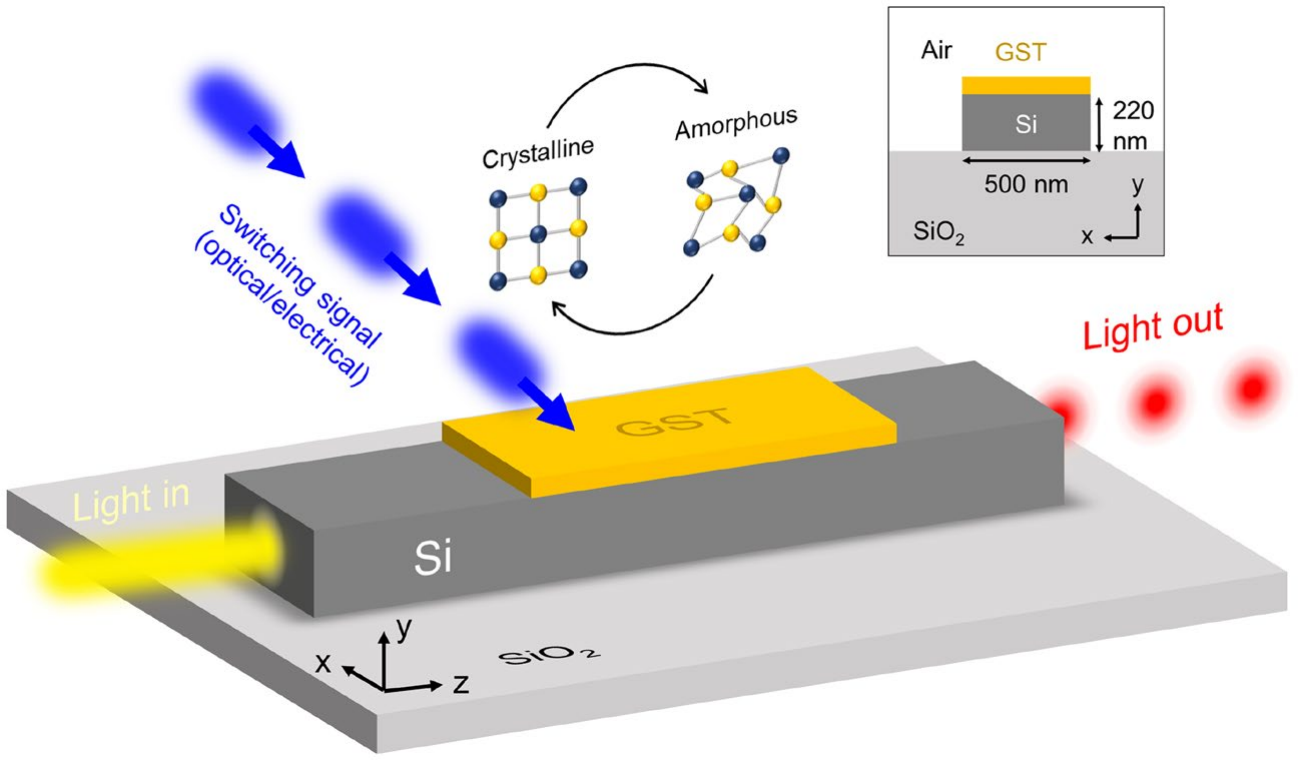
Illustration of a GST-loaded silicon waveguide and its working principle for optical switching. The GST switches between crystalline and amorphous states upon an optical or electrical switching signal. Between both states, the refractive index of the GST suffers a dramatic change in both real and imaginary parts, resulting in an amplitude and phase modulation of the output light. Inset shows the cross-section and the dimensions of the GST/Si waveguide.

Figure 1 illustrates the working principle of the considered GST/Si waveguide used for optical switching. The waveguide comprises a standard silicon waveguide with a layer of GST on top (see inset of Fig. 1). Switching between amorphous and crystalline is achieved by triggering the phase change with an external or on-chip stimuli such as evanescent coupling or microheaters. For optical actuation, heating originates inside the GST patch, while for microheaters, the heat distribution depends on the heater design. Nevertheless, a full change in the GST patch is usually achieved by tailoring the shape of the optical or electrical pulses. The phase change is accompanied by a variation on both real and imaginary parts of the effective refractive index of the guided light. Therefore, at the output of the hybrid waveguide, the light is modulated in both phase and amplitude. On the other hand, the modulation strength is strongly dependent on the thickness of the GST patch, which can be tailored between a few and dozens of nanometers during the  fabrication process.

### Optical modes coupling and propagation

**Figure 2:**
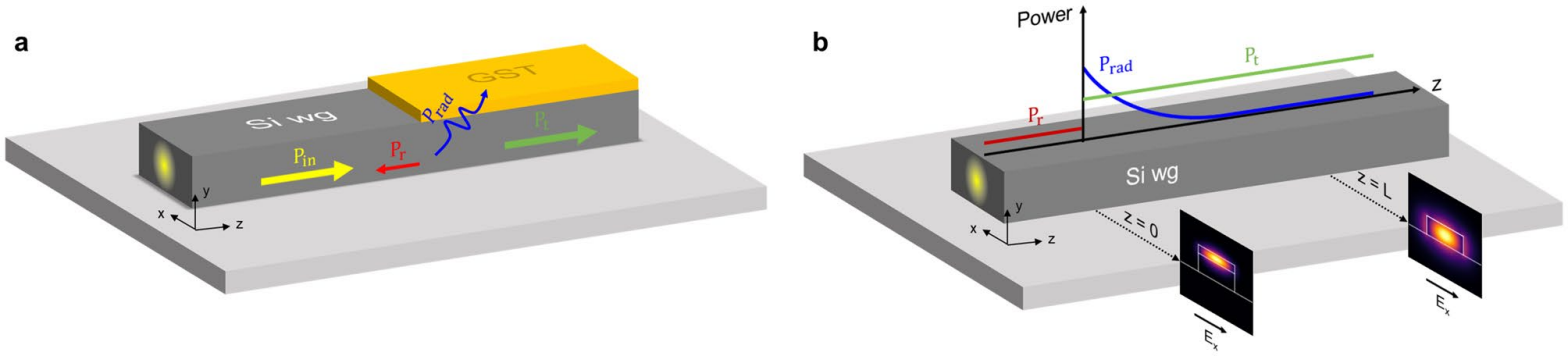
(**a**) Illustration of the power transfer between a silicon and GST/Si waveguide. Due to the optical mismatch between both waveguides, the incident power ($P_{in}$) is radiated outside the waveguide ($P_{rad}$), reflected ($P_{r}$), and transmitted ($P_{t}$). (**b**) Illustration of the technique used to obtain the coupling efficiency between the optical modes of the GST/Si waveguide and the fundamental TE mode of the silicon waveguide by exploiting the reciprocity property of the coupling process.

For the materials used in this work, we consider the complex refractive indices ($n+j\kappa$) given in the "Methods" section. Those values are used for our numerical simulations, which unless otherwise stated, are given at 1550 nm and for the transverse electric (TE) polarization. Figure 2a depicts the power transfer between a silicon and GST/Si waveguide in the presence of a step discontinuity. Because of the refractive index profile mismatch between both structures, several optical modes may be excited in the GST/Si waveguide to fulfill the field continuity condition at the interface. In this regard, the normalized transmitted electric field along the propagation direction (z-axis) in the GST/Si waveguide can be approximated as a linear combination of the different supported modes or eigenmodes^56^ as

1 $$\begin{aligned} E_{t}(z) \approx \sum _{n}^{} \Gamma _{n}\exp \left( -\dfrac{2\pi \kappa _{\text {eff},n}}{\lambda } z \right) , \end{aligned}$$

where $\Gamma _{n}$ is the coupling coefficient between the optical mode of the silicon waveguide and the n$^{th}$-mode of the hybrid waveguide, $\kappa _{\text {eff},n}$ is the corresponding effective extinction coefficient, and $\lambda$ is the working wavelength.

To determine the value of $\Gamma _{n}$, we exploited the reciprocity of the coupling process. Hence, we calculated the coupling by exciting the silicon waveguide with the supported optical modes of the GST/Si waveguide. Figure 2b illustrates this process when a GST/Si mode excites the silicon waveguide. Optical mismatch gives rise to uncoupled power comprised by reflection and radiation. In this manner, $\Gamma _{n}$ can be obtained by assessing the amount of power that is transmitted, i.e., coupled to the TE mode of the silicon waveguide. To discriminate between radiated and transmitted power, we use the overlap integral:

2 $$\begin{aligned} \Gamma _{n}^{2} = \dfrac{\left| \int \mathbf{E }_{n}\times \mathbf{H }_{in} dS \right| ^{2}}{\ \int \mathbf{E }_{n} \times \mathbf{H }_{n} dS \int \mathbf{E }_{in} \times \mathbf{H }_{in} dS}, \end{aligned}$$

where $\mathbf{E }/\mathbf{H }_{n}$ is the field profile in the silicon waveguide at a certain length when is excited with the n$^{th}$ GST/Si optical mode, and $\mathbf{E }/\mathbf{H }_{in}$ is the TE fundamental mode of the silicon waveguide. Simulation details can be found in the "Methods" section.

**Figure 3:**
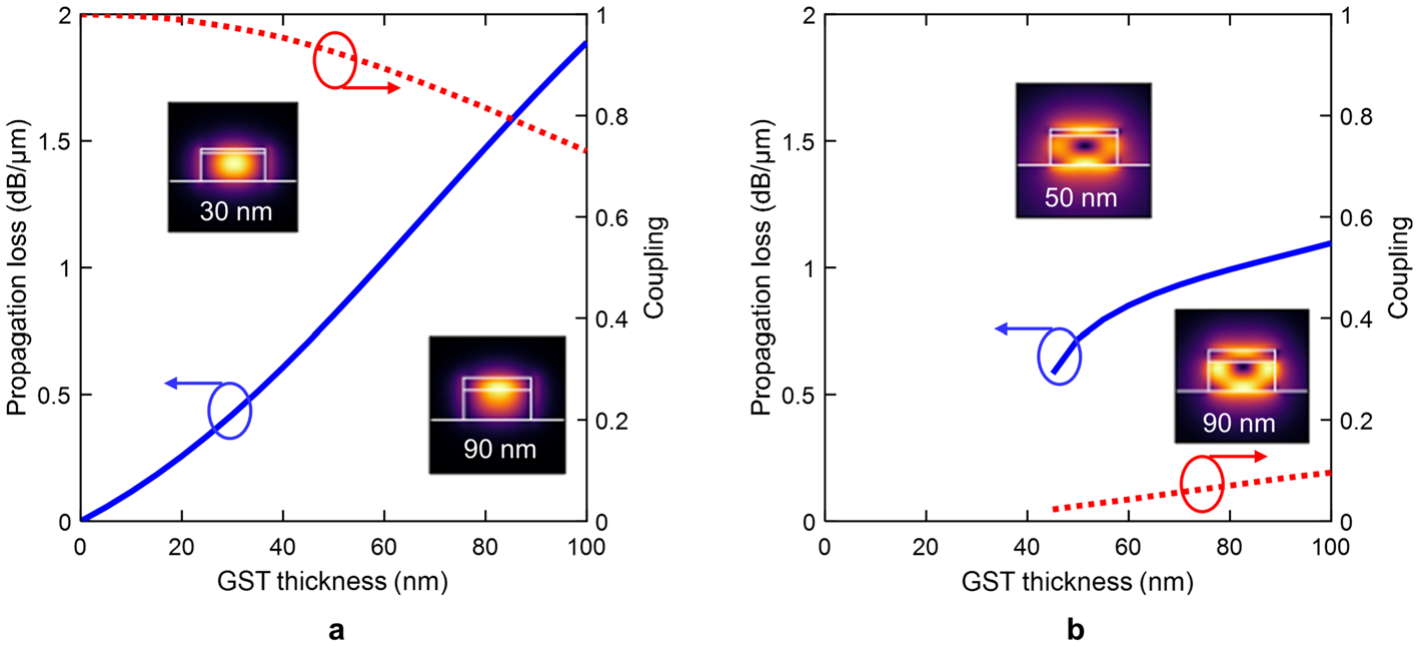
GST amorphous. Propagation losses (solid line) and coupling (dotted line) for the two supported optical modes: (**a**) fundamental GST TE-like (mode 0) and (**b**) hybrid (mode 1). Insets show the electric-field distribution |*E*| for a certain GST thickness.

Based on our simulations, the hybrid waveguide can support two optical modes (mode 0 and mode 1) in the amorphous state. Figure 3 shows the propagation losses and the associated power coupling coefficients as a function of the GST thickness. Mode 0 corresponds to the TE polarized fundamental mode of the GST/Si waveguide [Fig. 3a]. The light is mainly confined in the silicon waveguide for a very thin GST, giving rise to negligible coupling losses. As the GST thickness increases, the light is pushed up towards the GST [see insets of Fig. 3a]. However, the real part of the GST refractive index in the amorphous state is not high enough to allow guiding inside the GST patch. Consequently, a high percentage of the light remains in the silicon, and the optical coupling is still high. On the other hand, mode 1 corresponds to a hybrid optical mode with $E_{x}$ and $E_{y}$ components that has a cut-off around a thickness of 45 nm [Fig. 3b]. Although the propagation loss of this mode can be comparable to mode 0, the coupling is very weak since the light is mostly localized near the boundaries of the GST/Si waveguide.

**Figure 4:**
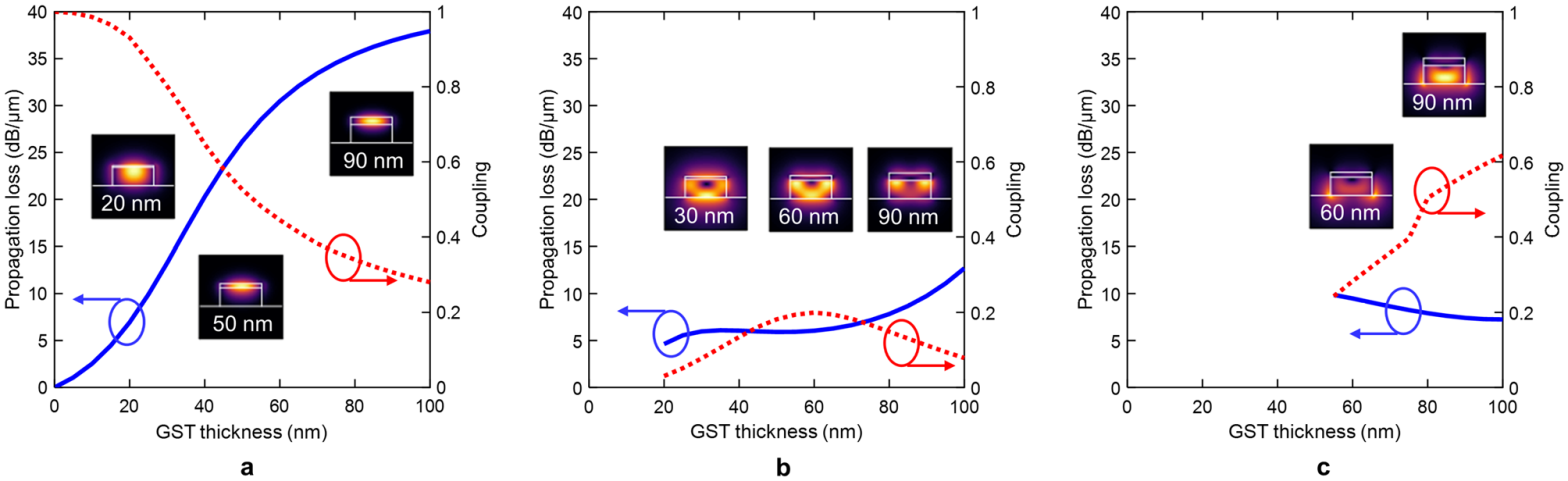
GST crystalline. Propagation losses (solid line) and coupling (dotted line) for the three supported optical modes: (**a**) fundamental GST TE-like (mode 0), (**b**) hybrid (mode 1), and (**c**) Si TE-like (mode 2). Insets show the electric-field distribution |*E*| for a certain GST thickness.

For the crystalline state, the real part of the GST refractive index suffers a dramatic increase giving rise to additional higher-order optical modes [see Fig. 4]. The propagation loss of the fundamental mode (mode 0) can reach almost 40 dB/$\upmu$m for the highest thickness of GST [see Fig. 4a]. However, the coupling varies from near-perfect coupling to a high coupling loss for thickness values between 20 nm and 60 nm. Such a change arises because the GST becomes the core of the hybrid waveguide [see insets of Fig. 4a]. The hybrid mode (mode 1) has a lower cut-off thickness of around 20 nm [see Fig. 4b] in contrast to the amorphous state. This reduction is due to the larger refractive index contrast. The propagation loss is between 5 and 15 dB/$\upmu$m with a maximum coupling of $\sim 0.2$. On the other hand, mode 2 has a cut-off thickness of around 55 nm [see Fig. 4c]. For this case, the light is guided through the silicon waveguide resembling the TE-like mode of a silicon waveguide [see insets of Fig. 4c]. Consequently, there is a weak interaction between the light and GST layer, yielding to the highest coupling values and lowest propagation losses among the three optical modes.

**Figure 5:**
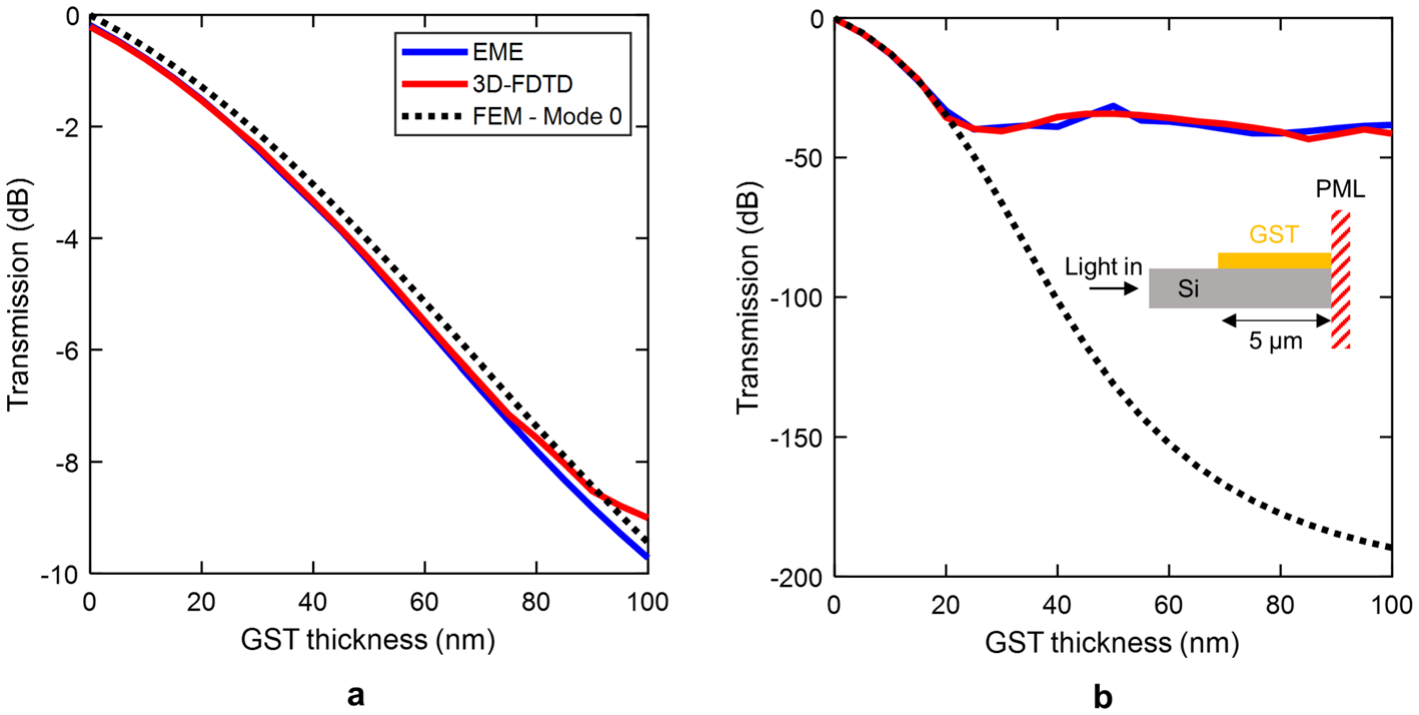
Comparison between 3D-FDTD and eigenmode expansion (EME). Transmission of GST/Si waveguide as a function of the GST thickness in the (**a**) amorphous and (**b**) crystalline state. Simulations were carried out for a step discontinuity between a silicon and GST/Si waveguide. The fundamental TE mode of the silicon waveguide impinges into a GST/Si waveguide ending in perfectly matched layer (see inset). Results were obtained for a 5 $\upmu$m-long-GST/Si waveguide. The dotted line stands for the transmission considering a perfect coupling of the fundamental (mode 0) TE mode into the GST/Si waveguide.

To verify the excitation and optical properties of such higher-order modes, we simulated the optical transmission between a silicon and GST/Si waveguide by using 3D-FDTD. Figure 5 shows the results obtained by 3D-FDTD (red line) and eigenmode expansion (EME) (blue line) using Eq. (1) together with values of Figs. 3 and 4. We considered a 5-$\upmu$m-long GST/Si waveguide like the shown in Fig. 2a ending in perfectly matched layer to avoid reflections [see inset of Fig. 5b]. The theoretical transmission considering only the propagation loss of the fundamental mode (mode 0) of the GST/Si waveguide is plotted for comparison (dotted line). Results show very good agreement between 3D-FDTD and EME simulations. Few discrepancies exist in the amorphous state since the fundamental GST/Si mode has small coupling losses and the propagation loss of the hybrid mode is similar to the fundamental (see Fig. 3). For the crystalline state, the influence of higher-order modes is notably for thicknesses higher than 20 nm. For lower values, modes 1 and 2 are cut-off and cannot be excited. As the GST patch is thickened, the coupling to the fundamental mode is reduced, and the values of modes 1 and 2 are increased (see Fig. 4). Higher-order modes have significantly lower propagation loss than the fundamental. Therefore, the discrepancies between the multimode behavior of the GST/Si waveguide and the single-mode assumption are enlarged.

### Selection of optimal thickness

For optical switching purposes, the optimal thickness of the GST should maximize the figure of merit (FOM) that relates the extinction ratio (ER), the insertion loss (IL), and the length of the hybrid waveguide, i.e., FOM = ER/(IL$\times$Length). For GST-loaded silicon waveguides, the insertion loss and extinction ratio depend on the GST length due to the multimode behavior of the hybrid waveguide in the crystalline state. On the other hand, ultra-compact waveguides are desired since this feature enables higher chip density and reduces the energy consumption.

**Figure 6:**
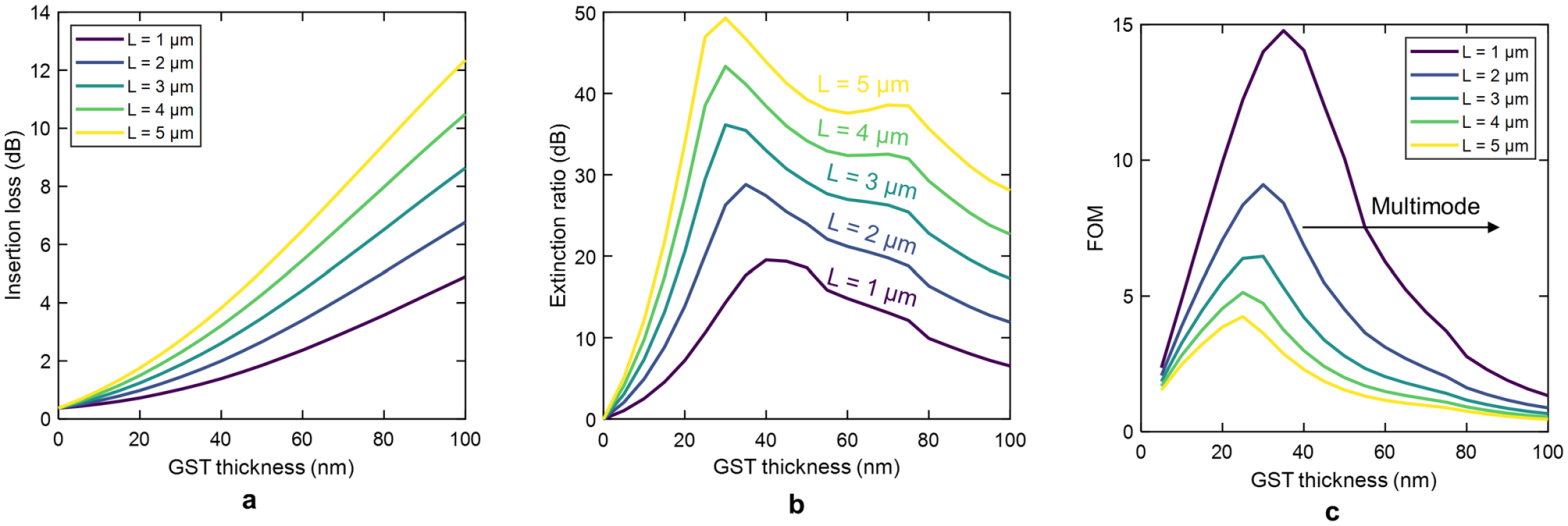
Optical performance of GST-loaded silicon waveguides calculated by eigenmode expansion as a function of the GST thickness and for different lengths. (**a**) Insertion loss. (**b**) Extinction ratio. (**c**) Figure of merit.

Figure 6 shows the optical performance (IL, ER, and FOM) of GST-loaded silicon waveguides calculated by EME as a function of the GST thickness and for different lengths. The insertion losses follow a similar trend like the propagation loss of the fundamental mode in the amorphous state due to its high coupling efficiency [see Fig. 3a]. Conversely, the response of the extinction ratio exhibits a maximum of around 30–40 nm, which stems from the multimode operation of the GST/Si waveguide. As the thickness of the GST increases, the contribution of the fundamental mode to total optical losses is reduced. These are mainly determined by the high-order modes (modes 1 and 2), which have much lower propagation losses than mode 0 (see Fig. 4). Such a response is transferred to the FOM, as shown in Fig. 6c. For thicknesses greater than 30-40 nm, the FOM suffers a dramatic reduction as the GST thickness increases.

### Experimental characterization

**Figure 7:**
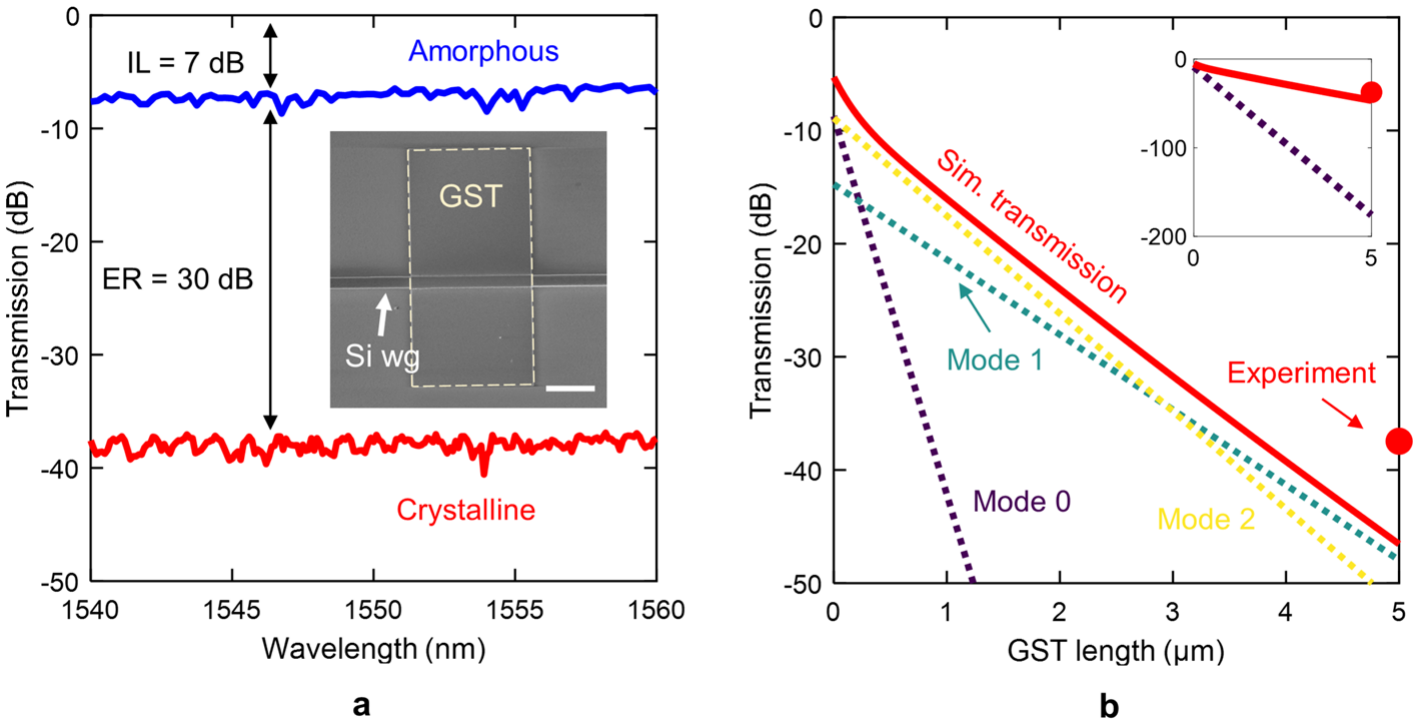
(**a**) Measured spectrum of the fabricated 70-nm-thick and 5-m-long GST/Si waveguide in the amorphous and crystalline state. The inset shows a scanning electron microscope (SEM) image of the waveguide. The scale bar is 2 $\upmu$m. The dashed line encloses the GST layer. (**b**) Simulated transmission and contribution of each mode along the 5-$\upmu$m-long GST/Si waveguide at a wavelength of 1550 nm. The experimental transmission value is also shown. The inset depicts a zoom-out of the plot.

We experimentally validated our simulations by fabricating and characterizing a multimode GST-loaded silicon waveguide in the amorphous and crystalline states (see "Methods" section for details about fabrication and characterization setup). We chose 70 nm for GST thickness to ensure the multimode operation in the GST/Si waveguide. Figure 7a shows the measured transmission spectrum of a 5-$\upmu$m-long GST/Si waveguide in the amorphous and crystalline state. The inset shows the fabricated waveguide’s scanning electron microscope (SEM) image. Results were normalized with respect to a silicon waveguide without GST. We obtained high insertion loss ($\sim$7 dB) and extinction ratio ($\sim 30$ dB), resulting in a FOM value of around 4. These results are in good agreement with our simulations describing the multimode operation (see Fig. 6). Accordingly, the optical performance is in the crystalline state is not dominated by the propagation loss of the fundamental mode but by the higher-order modes. Otherwise, the extinction ratio would have drastically increased to values above 100 dB, i.e., the measured power would have been limited by the noise floor.

To give a better insight into the multimode performance of the waveguide in the crystalline state, we simulated the transmission by EME as a function of the GST length, as shown in Fig. 7b. The transmission of each GST/Si optical mode and the experimental value (dot) are plotted for comparison. Discrepancies between simulation (solid red line) and experiment might be attributed to slight variations on the refractive index of the on-chip GST in the crystalline state compared to the thin film or a non-uniform crystallization of the GST layer. Nevertheless, as it can be noticed, the contribution of the fundamental mode (mode 0) is very small, mainly due to the extremely high propagation losses, and being negligible after a few nanometers [see inset of Fig. 7b]. Therefore, the transmission response of the 5-$\upmu$m-long GST/Si waveguide stems from the interplay between the high-order modes (modes 1 and 2), which have much lower propagation losses in comparison with the fundamental mode (mode 0).

## Discussion

In conclusion, we have investigated and characterized the impact of GST thickness on GST-loaded silicon waveguides for optimal optical switching. Our results show that for thicknesses greater than $\sim 30$ nm, GST/Si waveguides may work in a multimode regime arising from the large refractive index of GST. The multimode operation results in a reduction of the performance compared to the assumption of single-mode transmission, in which the light is mostly guided within the GST layer. Through numerical simulation, we unveil that the origin of such a multimode is the large step-index discontinuity between the silicon and GST/Si waveguide. Our experimental results confirm the optical performance’s multimode operation and the associated turndown. For a 70-nm-thick and 5-$\upmu$m-long GST patch, we obtain an insertion loss and extinction ratio of 7 dB and 30 dB, respectively, resulting in a FOM of around 4. Such values represent almost a six-fold reduction in the FOM value with respect to the assumption of single-mode operation. Hence, confirming that utilizing thick GST layers may not be optimal in GST-loaded silicon waveguides for optical switching purposes.

Our results are helpful for further developing and optimizing GST-based silicon photonic components and circuits that might apply in emerging fields such as non-volatile switching, photonic tensor cores, and neuromorphic computing.

## Methods

### Optical constants and GST refractive index characterization

For the materials used in this work, we consider the complex refractive indices ($n+j\kappa$) shown in Table 1. For GST, we experimentally determined the refractive index in both states from a 70-nm-thick Ge$_{2}$Sb$_{2}$Te$_{5}$ thin film deposited in a gold substrate by using e-beam evaporation. As-deposited GST was amorphous, while crystallization was achieved by heating the sample with a hot plate at $\sim 180^{\circ }$C for 10 min. The complex refractive index was calculated from reflection measurements using Fourier-transform infrared (FTIR) spectroscopy.

**Table 1:** Refractive index of the materials at 1550 nm. a-GST (c-GST) stands for GST in the amorphous (crystalline) state.

	Air	Si	SiO$_{2}$	a-GST	c-GST
$n+j\kappa$	1	3.476	1.444	$4.373+j0.0937$	$6.463 + j1.074$

### Simulation of the optical modes and coupling

The optical modes and their associated effective complex refractive indices were obtained by using 2D finite element method (FEM) using FemSIM tool from RSoft^57^. We applied a non-uniform mesh of 20 nm $\times$ 20 nm (x $\times$ y) with a minimum division of 10 points in the GST thickness (y-axis). On the other hand, the mode coupling was obtained by 3D finite-difference time-domain (3D-FDTD) simulations using FullWAVE tool from RSoft^58^. We applied a non-uniform mesh of 20 nm $\times$ 20 nm $\times$ 20 nm (x $\times$ y $\times$ z) with a minimum division of 10 points in the thickness (y-axis) of the GST layer. Perfectly matched layers (PMLs) were utilized to all the boundaries, except at $x=0$ in which symmetric boundary condition was used.

### Fabrication and characterization setup

The photonic strucrures were fabricated in 220-nm-thick silicon-on-insulator (SOI) and patterned by e-beam. GST was deposited by e-beam evaporation from a Ge$_{2}$Sb$_{2}$Te$_{5}$ source and etched by lift-off in acetone. As-deposited GST was amorphous, while crystallization was achieved by heating the samples above the crystallization temperature ($\sim 180^{\circ }$C) for several minutes. Grating couplers were used for fiber-to-chip coupling.

To characterize the sample, we used a continuous-wave tunable laser working in the C-band (Photonetics ECL-1600). Polarization was adjusted to TE with a polarization controller (Thorlabs FC032) before injecting it into the chip. At the output, optical power was recorded using a power meter (Thorlabs PM320E) and a high-sensitivity photodiode (Thorlabs S155C).

## Data Availability

The datasets generated and/or analyzed during the current study are available in the Zenodo repository, 10.5281/zenodo.6201510.
